# Differential Item Functioning in the SF-36 Physical Functioning and Mental Health Sub-Scales: A Population-Based Investigation in the Canadian Multicentre Osteoporosis Study

**DOI:** 10.1371/journal.pone.0151519

**Published:** 2016-03-21

**Authors:** Lisa M. Lix, Xiuyun Wu, Wilma Hopman, Nancy Mayo, Tolulope T. Sajobi, Juxin Liu, Jerilynn C. Prior, Alexandra Papaioannou, Robert G. Josse, Tanveer E. Towheed, K. Shawn Davison, Richard Sawatzky

**Affiliations:** 1 Department of Community Health Sciences, University of Manitoba, Winnipeg, MB, Canada; 2 School of Health and Human Performance, Dalhousie University, Halifax, NS, Canada; 3 Department of Public Health Sciences, Queen’s University, Kingston, ON, Canada; 4 Division of Clinical Epidemiology, McGill University Health Centre, Montréal, QC, Canada; 5 Department of Community Health Sciences & O’Brien Institute for Public Health, University of Calgary, Calgary, AB, Canada; 6 Departmentof Mathematics and Statistics, University of Saskatchewan, Saskatoon, SK, Canada; 7 Medicine and Endocrinology, University of British Columbia, Vancouver, BC, Canada; 8 Departmentof Medicine, McMaster University, Hamilton, ON, Canada; 9 Departmentof Medicine, Faculty of Medicine, University of Toronto, Toronto ON, Canada; 10 Department of Medicine, Queen’s University, Kingston, ON, Canada; 11 Facultyof Graduate Studies, University of Victoria, Victoria, BC, Canada; 12 School of Nursing, Trinity Western University &Centre for Health Evaluation and Outcomes Sciences, Providence Health Care, Langley, BC, Canada; University of Vienna, School of Psychology, AUSTRIA

## Abstract

**Background:**

Self-reported health status measures, like the Short Form 36-item Health Survey (SF-36), can provide rich information about the overall health of a population and its components, such as physical, mental, and social health. However, differential item functioning (DIF), which arises when population sub-groups with the same underlying (i.e., latent) level of health have different measured item response probabilities, may compromise the comparability of these measures. The purpose of this study was to test for DIF on the SF-36 physical functioning (PF) and mental health (MH) sub-scale items in a Canadian population-based sample.

**Methods:**

Study data were from the prospective Canadian Multicentre Osteoporosis Study (CaMos), which collected baseline data in 1996–1997. DIF was tested using a multiple indicators multiple causes (MIMIC) method. Confirmatory factor analysis defined the latent variable measurement model for the item responses and latent variable regression with demographic and health status covariates (i.e., sex, age group, body weight, self-perceived general health) produced estimates of the magnitude of DIF effects.

**Results:**

The CaMos cohort consisted of 9423 respondents; 69.4% were female and 51.7% were less than 65 years. Eight of 10 items on the PF sub-scale and four of five items on the MH sub-scale exhibited DIF. Large DIF effects were observed on PF sub-scale items about vigorous and moderate activities, lifting and carrying groceries, walking one block, and bathing or dressing. On the MH sub-scale items, all DIF effects were small or moderate in size.

**Conclusions:**

SF-36 PF and MH sub-scale scores were not comparable across population sub-groups defined by demographic and health status variables due to the effects of DIF, although the magnitude of this bias was not large for most items. We recommend testing and adjusting for DIF to ensure comparability of the SF-36 in population-based investigations.

## Introduction

Self-report health status measures, like the Short Form 36-item Health Survey (SF-36) [1], can provide rich information about the overall health of a population [2,3] and its components, such as physical, mental, and social health. However, in order for comparisons of health status across population sub-groups to be accurate, these self-report measures must be valid and reliable. Construct validity and test-retest reliability are frequently evaluated for a measure’s summary score(s), that is, after the item responses have been summed [4]. Self-report measures are less often evaluated for the effects of differential item functioning (DIF), which can also affect construct validity [5]. DIF occurs when individuals with the same underlying (i.e., latent) level of health do not interpret a measure’s items in the same way. DIF can result in an unexpected lack of scale comparability and erroneous conclusions about the presence of group differences [5].

The SF-36 has undergone comprehensive psychometric evaluations of its reliability and validity [6–9]. DIF and related topics of differential scale functioning have been investigated for the SF-12 and SF-36 [10–16], but most of these analyses have been conducted in clinical or disease-specific samples. DIF analyses are often conducted on demographic and ethnic characteristics even though other determinants of health, including risk factors for poor health and presence of chronic conditions may be potential sources of DIF [11,13,17].

The physical functioning (PF) and mental health (MH) sub-scales of the SF-36 are the sub-scales most frequently investigated in psychometric evaluations, and are also commonly used sub-scales to compare health status at the population level. Our study objective was to test for DIF on the SF-36 PF and MH sub-scale items in population-based data on demographic and health-related variables.

## Methods

### Data Source

Study data were from the Canadian Multicentre Osteoporosis Study (CaMos), a prospective cohort study initiated to provide national prevalence and incidence estimates for osteoporosis and osteoporosis-related fractures in the Canadian population [18]. Baseline data, which were the focus of the current study, were collected in 1996–1997, using both personal interview and papeer-based questionnaires, from participants in nine Canadian regional urban centres. Respondents were at least 25 years of age and were recruited without regard for disease status. Details of the methodology to select the CaMos cohort and collect the study data have been described elsewhere [2,18]. To ensure the quality and integrity of the data, interviewers are trained to minimize the amount of missing data (i.e., probe for responses), and respondents are re-contacted if clarification of responses is required to resolve inconsistencies in the data.

### Measures

Version 1 of the SF-36 was used in CaMos; it encompasses eight sub-scales: PF, role physical, bodily pain, general health, vitality, social functioning, role emotional, and MH. Item responses are captured using dichotomous or ordinal scales [1]. The PF sub-scale contains 10 item, each having three response options: limited a lot, limited a little, and not limited at all. The MH sub-scale consists of five items, each having six response options: all of the time, most of the time, a good bit of the time, some of the time, a little of the time, none of the time. Responses for “Have you felt calm and peaceful?” and “Have you been a happy person?” are reverse coded so that higher scores represent better MH, in keeping with the other sub-scale items.

DIF analyses were conducted for the following demographic and health-related variables: sex, age group, body weight status, and self-perceived general (i.e., overall) health. Age was classified as 25–49 years, 50–64 years, 65–74 years, and ≥75 years. Body weight status was based on BMI, which was calculated from measured height and weight (kg/m^2^), and was categorized as under or normal weight (<25.0), overweight (25.0–29.9) and obese (≥30.0) in accordance with published guidelines [19]. General health was based on a single question in the SF-36 and was categorized as excellent/very good, good, and fair/poor.

### Analysis

The analyses were conducted for respondents with complete information on all items or explanatory variables. Descriptive analyses were conducted using frequencies and percentages.

Both non-parametric and parametric approaches have been proposed to test for DIF; they can accommodate multiple population characteristics (i.e., covariates) that may be associated with item responses. Parametric approaches include logistic regression analysis [20], the multiple indicators multiple causes (MIMIC) model, and item response theory (IRT) models [5,21,22], with the latter two being popular because they can be applied to binary and ordinal item responses [5], are flexible to incorporate one or more latent constructs, and can be readily implemented using existing software. Woods [23] demonstrated, via simulation, that the MIMIC model will produce more accurate results than the IRT model, in a simple two-group scenario, for small group sizes.

**Fig 1:**
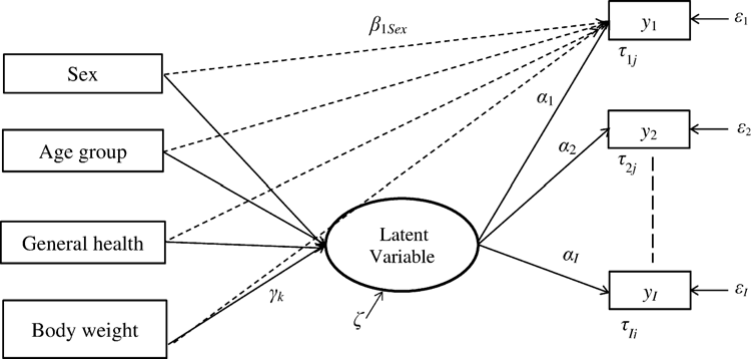
Illustration of the multiple indicators multiple causes model to test for differential item functioning on SF-36 sub-scale items. In this model, *y*_*i*_ is the *i*th sub-scale item (*i* = 1, …, *I*); the dashed arrow from each covariate to the item represents the DIF (i.e., direct) effect; *β*_*1Sex*_ is the regression coefficient for the difference in thresholds on item *i* for males and females; similar regression coefficients are defined for other model covariates; *α*_*i*_ is the regression coefficient for the latent variable and the *i*th item; *γ*_*k*_ is the regression coefficient for the latent variable and the *k*th covariate (*k* = 1, …, *K*); *τ*_*ij*_ is the threshold for the (*j*– 1) th response category *(j* = 1, *…*,*J*) for item *i*; *ε*_*i*_ is the error term for the *i*th item; *ζ* is the residual error for the latent variable.

We adopted the MIMIC model and used the following strategy to test for DIF. First, the assumption of unidimensionality, that is, that all sub-scale items measure a single construct [5], was examined by applying factor analysis with oblique rotation to the polychoric correlations for the sub-scale items [24]. The first eigenvalue should be significantly higher than the second eigenvalue (i.e., ratio > 4:1) to support unidimensionality [25]. As well, fit of a unidimensional measurement model was assessed using the the comparative fit index (CFI) and the root mean square error of approximation (RMSEA). A RMSEA value ≤0.10, and a CFI value >0.90 indicate acceptable model fit [23,26,27]. The measurement model defines the relationship between the underlying latent construct (i.e., PF or MH) and the sub-scale items, while a structural model is used to specify the relationships of the observed covariates with the sub-scale items and the latent construct [23,28,29]. A statistically significant direct effect of the observed covariates on the sub-scale item(s) indicates uniform DIF, after controlling for differences in latent health status between comparison groups (Fig 1). In keeping with previous research [28–31], a three-step procedure was adopted: (a) empirically select an anchor item(s) for the sub-scale, (b) test for DIF on each sub-scale item, and (c) fit a final model that allows for differential functioning on the individual sub-scale items.

At least one anchor item is needed to define the latent construct on which the groups are compared. We adopted the following method to select an anchor item [32]. First, a model was fit to the data that included the effects of the covariates on the latent variable but no direct effects between the covariates and the sub-scale items. Next, a series of models were fit to the data that added paths from the covariates to the items; this was done one sub-scale item at a time. A likelihood ratio (LR) statistic was used to test the difference between the two models for each item [32]. An item was labelled DIF-free if the LR statistic was not statistically significant. The DIF-free item with the smallest LR statistic was selected as the anchor item. If none of the items were DIF-free, then the item with the smallest LR statistic was selected as the anchor item [32].

Next, an unconstrained DIF model was fit to the data that included direct effects from the covariates to each sub-scale item, except for the anchor item. Then we fit a second set of no-DIF models that did not contain direct effects. A LR statistic was used to test the difference between the constrained “no-DIF” model in which the covariate direct effects were set to zero and the unconstrained DIF model with freely-estimated covariate direct effects for each item. A statistically significant LR statistic indicates the presence of uniform DIF on the item.

The third step was to fit a model that included direct effects of the covariates on all the items for which DIF was identified in the previous step, as well as direct effects of the covariates on the latent variable. This final DIF model was used to obtain parameter estimates and predict the factor scores.

Regression coefficients from the final model were exponentiated to produce odds ratios (ORs) to estimate the size of the DIF effects. Cut-points of 0.3, 0.5, 0.7 for the log of the ORs were used to indicate small, moderate and large DIF effect sizes [33]. Accordingly, an OR outside the range of 0.5 to 2.0 (i.e., a large effect size) was used to indicate a clinically meaningful DIF effect [13,34].

The impact of DIF was also investigated by testing the associations of the demographic and health variables with the predicted factor scores in the final DIF and no-DIF models [13]. Differences in the predicted scores on the covariates for the two models were computed [13] and subsequently tested for statistical significance using a multivariable linear regression model.

Parameters were estimated using the maximum likelihood method with robust standard errors (MLR) [24]. Details for computation of the differences in log likelihood statistics for nested models can be found on the Mplus website (https://www.statmodel.com/chidiff.shtml). Given the increased probability of a Type I error when conducting multiple tests of significance, we adopted the Bonferroni procedure for all inferential analyses [35], adjusting the nominal level of significance (i.e., *α*) by either the number of items in the PF or MH sub-scales or the number of levels of the covariates, depending on the analysis.

The MIMIC framework was implemented using Mplus version 7.11 [24], while SAS version 9.3 was used to conduct the descriptive analyses [36]. To identify each model in the MIMIC framework, the latent factor mean was constrained to zero and the variance was constrained to one. This research was approved by the University of Manitoba Health Research Ethics Board. CaMos participants provided written informed consent at the time of study entry.

## Results

Of the 9423 respondents in the CaMos cohort, 69.4% were women and slightly more than half (51.7%) were under 65 years of age. One third reported being in good health and 11.0% reported being in fair or poor health. Overweight and obese individuals accounted for 40.7% and 22.3% of respondents, respectively. A total of 96.2% (*n* = 9062) were retained in the PF sub-scale analysis and 96.7% (*n* = 9115) were retained in the MH sub-scale analysis after excluding individuals with missing observations.

Table 1 shows the distribution of item responses for the PF and MH sub-scales. Close to half (43.3%) of respondents reported being limited a lot in vigorous activities, while only 16.0% were limited a lot in walking more than a mile. For the MH sub-scale, few respondents reported being very nervous, feeling so down in the dumps that nothing could cheer them up, or feeling downhearted and blue for some or all of the time. S1 Table and S2 Table shows the percentage of respondents for selected categories of the PF and MH sub-scale items on each of the covariates. There was a substantial increase in the percentage of respondents who experienced a lot of limitations in their activities, including walking a single block, with age. While there were few differences in the PF item percentages on “a lot of limitations” between respondents who were normal or underweight and overweight, those who were obese had a number of limitations. Respondents with excellent or very good overall health had substantially fewer functional limitations and were also much more likely to feel calm and peaceful and be happy. There were fewer differences for the MH sub-scale items, except for general health status.

**Table 1:** Percentage distribution of item responses on the SF-36 physical functioning and mental health sub-scale items in the Canadian Multicentre Osteoporosis Study.

Item	Response options					
**Physical functioning (*N* = 9062)**	**Limited a lot**		**Limited a little**		**Not limited at all**	
PF1: Vigorous activities	43.3		34.7		22.0	
PF2: Moderate activities	12.2		24.4		63.4	
PF3: Lifting or carrying groceries	8.5		22.2		69.3	
PF4: Climbing several flights of stairs	14.7		29.8		55.4	
PF5: Climbing one flight of stairs	5.2		15.7		79.1	
PF6: Bending, kneeling or stooping	12.6		33.1		54.4	
PF7: Walking more than a mile	16.0		20.7		63.3	
PF8: Walking several blocks	10.3		14.9		74.8	
PF9: Walking one block	3.6		9.9		86.5	
PF10: Bathing or dressing self	1.7		6.3		92.0	
**Mental health (*N* = 9115)**	**All of the time**	**Most of the time**	**Good bit of the time**	**Some of the time**	**Little of the time**	**None of the time**
*Have you…*						
MH1: Been a very nervous person?	1.3	3.1	5.4	17.3	29.9	43.1
MH2: Felt so down in the dumps that nothing could cheer you up?	0.3	1.0	1.7	7.3	17.1	72.5
MH3: Felt calm and peaceful?	10.6	46.4	18.4	15.6	5.9	3.2
MH4: Felt downhearted and blue?	0.3	1.5	3.4	17.6	35.1	42.1
MH5: Been a happy person?	18.4	54.9	13.1	8.6	2.8	2.2

For the PF sub-scale items, the factor analysis produced eigenvalues of 7.61 for the first factor and 0.63 for a second factor; the ratio of eigenvalues of 12.1 suggests a unidimensional factor structure. Further analysis revealed that a one-factor model without error covariances had a reasonable fit to the data (RMSEA = 0.11, 95% CI = 0.10–0.11; CFI = 0.98). However, a single-factor model with error covariances between five pairs of items (PF1 with PF2, PF2 with PF3, PF4 with PF5, PF7 with PF8, PF8 with PF9) resulted in a better fit to the data (RMSEA = 0.057, 95% CI = 0.054–0.067, CFI = 0.99). Factor loadings for the sub-scale items ranged from 0.80 to 0.93.

For the MH sub-scale items, factor analysis revealed that the eigenvalues were 3.13 for the first factor and 0.69 for a second factor. The one-factor model produced CFI = 0.97 and RMSEA = 0.15 (95% CI = 0.14–0.15), and factor loadings ranged in value from 0.61 to 0.82. Both of these analyses support a single dominant factor for both the PF and MH sub-scale items.

In step 1 of the DIF analysis, which focused on identifying anchor items for each of the sub-scales, all of the LR statistics were statistically significant, indicating that no items were DIF-free. However, “walking more than a mile” and “felt so down in the dumps that nothing could cheer you up” had the smallest LR statistics and thus were selected as anchor items. Table 2 displays the LR test results for DIF. All the items except the anchor items showed statistically significant effects. The factor loadings and item thresholds are reported in S3 Table.

**Table 2:** Tests for differential item functioning on the SF-36 physical functioning and mental health sub-scale items in the Canadian Multicentre Osteoporosis Study.

Item	LL^a^	df^b^	p-value^c^
**Physical functioning (N = 9062)**			
PF1	200.76	8	**<0.0001**
PF2	105.79	8	**<0.0001**
PF3	182.35	8	**<0.0001**
PF4	45.50	8	**<0.0001**
PF5	23.24	8	**0.0031**
PF6	77.18	8	**<0.0001**
PF7	†	†	†
PF8	27.77	8	**0.0005**
PF9	50.08	8	**<0.0001**
PF10	47.40	8	**<0.0001**
**Mental health (*N* = 9115)**			
MH1	75.23	8	**<0.0001**
MH2	†	†	†
MH3	56.53	8	**<0.0001**
MH4	24.05	8	**0.0023**
MH5	41.56	8	**<0.0001**

^a^LL = log of the likelihood function.

^b^df = degrees of freedom. LL values and df were computed as the difference between the unconstrained DIF model (i.e., including DIF effects for all items except the anchor item) and the constrained DIF model (i.e., excluding DIF effects for one item at a time).

^c^All p-values are statistically significant using a Bonferroni-corrected *α* = .05/9 = .0056 for the physical functioning sub-scale items and *α* = .05/4 = .0125 for the mental health sub-scale items.

† = anchor item.

As Table 3 reveals, eight of the 10 PF sub-scale items exhibited DIF by age group, sex, body weight status and/or self-perceived health status. Women had a greater odds of reporting limitations on PF2, PF3, and PF4, and a greater odds of having no limitations in bathing or dressing (PF10; OR = 1.66), even after controlling for differences in their underlying functional abilities. Older respondents with the same underlying functional abilities scored lower than younger respondents in vigorous and moderate activities, and scored higher in walking one block and bathing/dressing self. With respect to self-perceived general health status, DIF effects were observed for four PF items, with respondents in poorer health being more likely to endorse limitations in vigorous and moderate activities and also being more likely to report better physical function in walking one block, relative to those in very good or excellent health, after controlling for differences in latent physical functioning. Relative to normal/underweight respondents, overweight and obese people were more likely to report fewer limitations in moderate activities and lifting or carrying groceries, but were more likely to report limitations in bending, kneeling or stooping, after controlling for overall physical health status. The DIF effects were small to large in size across the PF items; the smallest effects were observed for the following PF items: vigorous activities and lifting and carrying groceries. The largest effects were observed for lifting and carrying groceries and walking one block.

**Table 3:** Odds ratios for differential item functioning (i.e., direct) effects on the SF-36 physical functioning and mental health sub-scale items in the Canadian Multicentre Osteoporosis Study.

Item	Sex^a^	Age Group			General Health		Body Weight	
	Female	50–64	65–74	≥75	Good	Fair/Poor	Overweight	Obese
**Physical functioning**^b^								
PF1	0.86	**0.56**	**0.34**	**0.24**	**0.73**	**0.46**	1.04	1.10
PF2	**0.71**	0.80	0.89	0.73	0.82	**0.61**	**1.43**	**2.28**
PF3	**0.39**	0.87	1.14	1.13	0.82	0.67	**1.41**	**2.46**
PF4	**0.65**	0.89	1.06	1.05	0.83	0.80	0.79	0.81
PF5	0.88	1.00	1.09	1.62	0.98	1.16	0.85	0.82
PF6	0.93	0.84	0.93	1.18	0.92	0.90	**0.72**	**0.57**
PF7	†^c^	†	†	†	†	†	†	†
PF8	1.19	0.99	1.27	1.67	1.22	1.52	1.13	1.14
PF9	1.40	1.59	**2.34**	**4.22**	1.31	**2.51**	1.03	1.10
PF10	**1.66**	1.51	**2.15**	**2.43**	1.12	0.90	0.95	1.12
**Mental health**^b^									
MH1	**0.80**	0.95	0.93	**1.28**	0.96	0.96	**1.29**	**1.57**
MH2	†	†	†	†	†	†	†	†
MH3	**0.70**	**1.26**	**1.36**	**1.49**	**0.82**	**0.63**	0.97	1.18
MH4	**0.80**	0.92	0.79	0.84	1.16	0.91	1.08	1.07
MH5	1.03	1.06	1.23	**1.48**	**0.74**	**0.63**	1.07	**1.26**

^a^Reference groups for the covariates are male, age 25–49 years, excellent/very good, underweight/normal weight.

^b^Bold odds ratios denote values that are statistically significant at *α* = 0.05/9 = 0.0056 for the physical functioning sub-scale items and *α* = 0.05/5 = 0.0125 for the mental health sub-scale items.

† = anchor item.

Four items in the MH sub-scale exhibited DIF effects. Women tended to endorse having been a very nervous person, feeling less calm and peaceful, and feeling downhearted and blue more often than men, after controlling for differences in underlying MH status. Older respondents, relative to younger respondents who had the same latent MH status showed a greater propensity to feel calm and peaceful, and to be happy. Respondents with good and fair/poor health had lower odds of feeling calm and peaceful, and being less happy relative to respondents with better self-perceived general health status, even after controlling for differences in underlying MH status. Respondents who were obese had greater odds of being very nervous. Obese individuals were more likely to endorse being happy people (OR = 1.26) even after controlling for differences in their latent MH status. However, the DIF effects were small to moderate in size for all MH sub-scale items.

Table 4 provides the regression parameter estimates for the effects of the covariates on the latent PF and MH scores in the DIF and no-DIF models, and their absolute differences. For the PF sub-scale, all of the covariates were associated with latent PF scores. However, adjustment for DIF resulted in a reduction in the size of the parameter estimates for all covariates, except for overweight. The magnitude of the change in the coefficients was greatest for the age groups (absolute differences ranged from 0.09 to 0.14) and sex (absolute difference 0.09). For the MH sub-scale, adjustment for DIF changed the direction of the association between body weight and MH. In the no-DIF model, MH scores were significantly higher for obese respondents relative to normal/underweight respondents. However, in the DIF model, MH scores were lower for obese respondents. The largest change in regression coefficients between no-DIF and DIF models was found for fair/poor health (absolute difference 0.12).

**Table 4:** Regression coefficient estimates for the effects of demographic and health status variables on physical functioning and mental health latent variables in models without differential item functioning (no-DIF) and with differential item functioning (DIF) in the Canadian Multicentre Osteoporosis Study.

Variable^a^	Physical functioning			Mental health		
	No-DIF	DIF	|*d|*^c^	No-DIF	DIF	|*d*|^c^
	Coeff. (SE)^b^	Coeff. (SE)		Coeff. (SE)	Coeff. (SE)	
**Female**	-0.34 (0.03)*	-0.25 (0.04)*	0.09*	-0.27 (0.03)*	-0.18 (0.04)*	0.09*
**Age Group**						
50–64	-0.60 (0.04)*	-0.46 (0.05)*	0.14*	0.21 (0.03)*	0.19 (0.05)*	0.02
65–74	-1.07 (0.04)*	-0.95 (0.05)*	0.12*	0.39 (0.04)*	0.36 (0.05)*	0.03*
≥75	-1.74 (0.05)*	-1.65 (0.06)*	0.09*	0.49 (0.04)*	0.40 (0.06)*	0.09*
**General Health**						
Good	-0.78 (0.03)*	-0.73 (0.04)*	0.05*	-0.59 (0.03)*	-0.54 (0.04)*	0.05*
Fair/Poor	-1.68 (0.04)*	-1.62 (0.05)*	0.06*	-1.16 (0.05)*	-1.04 (0.06)*	0.12*
**Weight Status**						
Overweight	-0.10 (0.03)*	-0.10 (0.04)*	0.00	0.03 (0.03)	-0.01 (0.04)	0.04*
Obese	-0.48 (0.03)*	-0.53 (0.04)*	0.05*	0.08 (0.03)*	-0.02 (0.04)	0.10*

^a^Reference groups for the covariates are male, age 25–49 years, excellent/very good, underweight/normal weight.

^b^Coeff. = coefficient; SE = standard error.

^c^**|***d***|** = absolute value of the difference in coefficient estimates between the no-DIF and DIF models.

*denotes values that are statistically significant at *α* = 0.05/8 = 0.00625.

As a sensitivity analysis, we fit separate models for each of the demographic and health status variables to test for DIF when the model was not simultaneously adjusted for all covariates. The ORs and magnitude of change in the coefficient estimates were similar to those reported in Tables 3 and 4.

## Discussion

This study revealed that the majority of the SF-36 PF and MH sub-scale items showed evidence of DIF across one or more of the investigated demographic and health status variables after controlling for differences in the the latent PF and MH of respondents in this population-based sample. Some of the DIF effects observed for the PF and MH sub-scale items were consistent with those found in previous studies [6,13,14]. Specifically, older people reported more limitations in vigorous and moderate activities, and fewer limitations in bathing or dressing, even after controlling for differences in their underlying PF [13,14]. Older respondents were also more likely to endorse feeling calm and peaceful than younger respondents after controlling for differences in their underlying MH [13,14] Women tended to identify more problems in lifting or carrying groceries and climbing several flights of stairs, whereas they reported fewer problems in bathing or dressing than men [6,13,14].

This study revealed that body weight status was associated with DIF; this effect was observed for three items on the PF sub-scale and two items on the MH sub-scale. However, the direction of the effect for body weight status was not consistent across all items on the PF sub-scale. Overweight/obese people may not perceive themselves as being limited in activities that occur on a daily basis, like lifting or carrying groceries, but may be limited in activities that are more likely to occur on a daily basis, such as bending, kneeling or stooping. One prior study that evaluated DIF on the SF-36 for BMI showed significant non-uniform DIF in vigorous PF activities by weight category [11]. The inconsistent effect and limited research on the potential for DIF by body weight status suggests an opportunity for further research, including an exploration of potential non-uniform DIF.

Adjustment for DIF did not change the direction of the associations between the covariates and the PF latent variables, but the strength of the associations did change such that they were almost always smaller in size after adjusting for DIF. This finding is consistent with other studies showing that significant sex and age differences in physical ability were not altered after adjusting for DIF [10,13]. The largest change in regression coefficients between the no-DIF and DIF models was observed for age on the PF sub-scale items. However, adjustment for DIF changed the association between body weight and MH. There was a significant difference in the latent MH variable between the obese and underweight/normal weight groups before DIF adjustment,whereas the difference became non-significant after controlling for DIF. Large changes in regression coefficients between the no-DIF and DIF models for MH were observed for fair/poor health and obese groups. The results suggest that the comparison of PF and MH scores across population sub-groups defined by demographic and health status variables may be biased if DIF is ignored. Group comparisons on PF may be most affected by age and sex, while group comparisons on MH may be most affected by body weight and general health status.

The advantages of the MIMIC framework over other methods for DIF detection, such as logistic regression or IRT models, are that it is based on well-established confirmatory factor analysis modeling processes and can be used to test for DIF on mutiple observed variables simultaneously. It can also be used to test for group differences in predicted factor scores simultaneously on mutiple observed covariates [13,37]. In addition, it allows for the analysis of DIF effects on a latent construct by comparing group differences in predicted factor scores between DIF-adjusted model and unadjusted models [13,37,38]. The MIMIC model for ordinal indicators is equivalent to the popular IRT graded-response model [22,38]; it is now frequently used to test for DIF and is known to perform well under a wide variety of data-analytic conditions [13,23,28,29]. Previous studies have demonstrated that the MIMIC model is effective in identifying DIF items, and is less sensitive to potential contamination of anchor items than other DIF detection methods [21,23,29]. Simulation studies have demonstrated that the MIMIC framework is more sensitive to identify DIF items and provides better control of Type I errors than conventional DIF test methods [21,31]. The chief disadvantage is that is cannot be used to test for non-uniform DIF (i.e., differential effects on item difficulty).

The study has other strengths. The DIF analysis was applied to a national population-based sample with a large sample size, which allowed for consideration of multiple demographic and health status variables. BMI was based on measured height and weight, and is therefore less susceptible to measurement error than self-reported height and weight. Age and sex are also confirmed by study staff during the data collection process, and are therefore likely to exhibit little, if any, measurement error.

However, this study is not without limitations. The MIMIC approach assumes that items have similar discriminative performance across comparison groups. Non-uniform DIF models, which test interactions between covariates and latent variables on the item responses, were not investigated because previous simulation studies have reported that the MIMIC approach may result in inflated Type Ι error rates when interaction terms are added to the model [39]. Performance of the MIMIC model for non-uniform DIF needs to be further studied. We only tested for DIF for two SF-36 sub-scales; other sub-scales have been examined for DIF using other statistical methods (i.e., logistic regression) [11,14]. We did not include other sub-scales in the current study; when there are small numbers of items (i.e., less than five items per subscale), the MIMIC framework is not an appropriate choice [13]. The CaMos sample includes a higher proportion of older adults and women than in the general population, thus the findings may under represent younger people (i.e., <50 years of age) and men. As well, for the one-factor model for the MH items, the RMSEA did not suggest a good fit to the data, although this finding is consistent with other studies [40,41]. Additional covariates could be considered in the model, although age and sex are two of the most common demographic variables considered in DIF analyses.

## Conclusions

In summary, this study revealed the presence of DIF in population-based SF-36 data. The results indicate that PF and MH sub-scale scores may not be comparable across sub-groups defined by demographic and health status variables without accounting for DIF. DIF can affect the validity of epidemiologic studies that use self-report measures of quality of life. Removing items that exhibit DIF affects the content validity of a measure, which is problematic for well-established and well-known measures such as the SF-36. Removing items can also affect the comparability of sub-scale scores across different studies [6,42]. An alternative strategy is to replace the items with equivalent items that do not exhibit DIF; item banks may be a resource for identifying DIF-free items [43]. However, often the preferred approach is to examine items for DIF prior to conducting other analyses and adjust for any identified DIF effects prior to making comparisons between sub-groups [6,11,42]. Evaluating population-based self-reported outcome measures for DIF, particularly on key demographic and health-related variables, should therefore be a routine component of all comparative analyses.

## Supporting Information

S1 Table Percentages of respondents for the category “Limited a lot” on the PF sub-scale items by demographic and health status variables in the Canadian Multicentre Osteoporosis Study (*n* = 9062). (DOCX)

S2 Table Percentages of respondents for the category “All/most/good bit of the time” on the MH sub-scale items by demographic and health status variables in the Canadian Multicentre Osteoporosis Study (*n* = 9115). (DOCX)

S3 Table Factor loading and item threshold estimates of the differential item functioning (DIF) model for the SF-36 physical functioning and mental health sub-scale items in the Canadian Multicentre Osteoporosis Study. (DOCX)
