# Supplementary material for: Differential Item Functioning in the SF-36 Physical Functioning and Mental Health Sub-Scales: A Population-Based Investigation in the Canadian Multicentre Osteoporosis Study
Source: PLoS One. 2016 Mar 21;11(3):e0151519. doi: 10.1371/journal.pone.0151519 (PMC4801323; doi:10.1371/journal.pone.0151519)
Supplement: S1 Table — (DOCX) [file pone.0151519.s001.docx]

**S1 Table. Percentages of respondents for the category “Limited a lot” on the PF sub-scale items by demographic and health status variables in the Canadian Multicentre Osteoporosis Study (*n* = 9062)**

| **Variables^a^** | **PF1** | **PF2** | **PF3** | **PF4** | **PF5** | **PF6** | **PF7** | **PF8** | **PF9** | **PF10** |
| --- | --- | --- | --- | --- | --- | --- | --- | --- | --- | --- |
| Male | 35.9 | 8.0 | 4.7 | 10.0 | 3.8 | 9.2 | 12.4 | 8.0 | 3.0 | 1.9 |
| Female | 47.6 | 14.9 | 11.0 | 17.6 | 6.4 | 14.7 | 18.5 | 12.1 | 4.5 | 2.1 |
| 25 – 49 years | 16.1 | 3.2 | 2.0 | 3.8 | 1.6 | 3.8 | 4.0 | 6.4 | 1.1 | 0.8 |
| 50 – 64 years | 32.6 | 7.6 | 4.9 | 9.2 | 2.6 | 8.9 | 9.1 | 13.0 | 1.6 | 1.1 |
| 65 – 74 years | 54.1 | 13.0 | 9.3 | 16.7 | 6.5 | 15.3 | 18.7 | 17.5 | 4.4 | 2.2 |
| 75+ years | 77.4 | 32.4 | 24.5 | 36.6 | 14.1 | 26.5 | 40.8 | 23.6 | 11.2 | 4.9 |
| Exc. or Very Good | 29.5 | 5.5 | 14.4 | 6.4 | 2.3 | 6.0 | 7.5 | 9.5 | 1.7 | 1.1 |
| Good | 56.2 | 14.7 | 30.7 | 19.7 | 6.1 | 16.1 | 28.7 | 20.4 | 4.2 | 2.0 |
| Fair/Poor | 82.9 | 44.6 | 37.8 | 47.6 | 21.3 | 39.7 | 51.7 | 28.0 | 15.2 | 7.0 |
| Normal or under weight | 40.5 | 13.07 | 8.9 | 12.4 | 4.4 | 9.0 | 14.3 | 9.6 | 3.4 | 1.6 |
| Overweight | 41.0 | 10.51 | 7.4 | 13.4 | 4.5 | 11.1 | 13.9 | 8.6 | 2.9 | 1.4 |
| Obese | 52.6 | 14.13 | 10.2 | 21.2 | 8.1 | 21.0 | 23.0 | 14.8 | 5.3 | 2.6 |

^a^For the complete list of items and response categories, please see Table 1.
