# Supplementary material for: Differential Item Functioning in the SF-36 Physical Functioning and Mental Health Sub-Scales: A Population-Based Investigation in the Canadian Multicentre Osteoporosis Study
Source: PLoS One. 2016 Mar 21;11(3):e0151519. doi: 10.1371/journal.pone.0151519 (PMC4801323; doi:10.1371/journal.pone.0151519)
Supplement: S2 Table — (DOCX) [file pone.0151519.s002.docx]

**S2 Table. Percentages of respondents for the category “All/most/good bit of the time” on the MH sub-scale items by demographic and health status variables in the Canadian Multicentre Osteoporosis Study (*n* = 9115)**

| **Variables^a^** | **MH1** | **MH2** | **MH3** | **MH4** | **MH5** |
| --- | --- | --- | --- | --- | --- |
| Male | 6.9 | 2.1 | 80.3 | 3.7 | 87.6 |
| Female | 11.1 | 3.6 | 72.9 | 6.1 | 85.5 |
| 25 – 49 years | 9.4 | 3.5 | 73.7 | 5.2 | 86.4 |
| 50 – 64 years | 10.2 | 3.1 | 75.8 | 5.0 | 86.1 |
| 65 – 74 years | 9.8 | 2.6 | 75.9 | 5.3 | 86.4 |
| 75+ years | 9.6 | 3.9 | 74.1 | 6.4 | 85.5 |
| Exc. or Very Good | 7.0 | 1.8 | 82.7 | 3.3 | 82.2 |
| Good | 11.2 | 3.1 | 69.9 | 5.1 | 65.7 |
| Fair/Poor | 20.4 | 9.8 | 51.9 | 16.2 | 49.4 |
| Normal or under weight | 10.9 | 2.9 | 75.6 | 4.6 | 86.5 |
| Overweight | 9.0 | 2.9 | 75.2 | 5.3 | 86.7 |
| Obese | 9.2 | 3.8 | 75.1 | 6.0 | 85.6 |

^a^For the complete list of questions and response categories, please see Table 1.
