# Supplementary material for: Differential Item Functioning in the SF-36 Physical Functioning and Mental Health Sub-Scales: A Population-Based Investigation in the Canadian Multicentre Osteoporosis Study
Source: PLoS One. 2016 Mar 21;11(3):e0151519. doi: 10.1371/journal.pone.0151519 (PMC4801323; doi:10.1371/journal.pone.0151519)
Supplement: S3 Table — (DOCX) [file pone.0151519.s003.docx]

**S3 Table. Factor loading and item threshold estimates of the differential item functioning (DIF) model for the SF-36 physical functioning and mental health sub-scale items in the Canadian Multicentre Osteoporosis Study.**

| **Item** | ***λ* (SE)^a^** | ***τ*_1_ (SE)^b^** | ***τ*_2_ (SE)** | ***τ*_3_ (SE)** |
| --- | --- | --- | --- | --- |
| **Physical functioning (*N* = 9062)** |  |  |  |  |
| PF1: Vigorous activities | 1.44 (0.04) | -3.61 (0.10) | -0.88 (0.09) |  |
| PF2: Moderate activities | 2.15 (0.06) | -7.35 (0.18) | -4.50 (0.15) |  |
| PF3: Lifting or carrying groceries | 2.19 (0.06) | -8.38 (0.20) | -5.35 (0.17) |  |
| PF4: Climbing several flights of stairs | 2.14 (0.06) | -7.21 (0.16) | -4.11 (0.14) |  |
| PF5: Climbing one flight of stairs | 2.61 (0.08) | -10.12 (0.27) | -7.01 (0.23) |  |
| PF6: Bending, kneeling or stooping | 1.67 (0.04) | -6.15 (0.14) | -3.17 (0.12) |  |
| PF7: Walking more than a mile | 2.94 (0.09) | -8.55 (0.24) | -5.73 (0.21) |  |
| PF8: Walking several blocks | 3.93 (0.16) | -12.03 (0.44) | -8.84 (0.37) |  |
| PF9: Walking one block^c^ | 4.31 (0.22) | -11.38 (0.55) | -- |  |
| PF10: Bathing or dressing self^c^ | 1.82 (0.08) | -5.80 (0.24) | -- |  |
| **Mental health (*N* = 9115)^d^** |  |  |  |  |
| MH1: Been a very nervous person | 1.28 (0.04) | -3.07 (0.08) | -1.50 (0.07) | 0.21 (0.07) |
| MH2: Felt so down in the dumps that nothing could cheer you up | 2.51 (0.09) | -6.56 (0.19) | -4.46 (0.15) | -2.31 (0.13) |
| MH3: Felt calm and peaceful | 1.58 (0.05) | -2.06 (0.09) | -0.82 (0.08) | 2.72 (0.09) |
| MH4: Felt downhearted and blue | 2.36 (0.07) | -5.68 (0.15) | -2.85 (0.11) | 0.001 (0.10)***** |
| MH5: Been a happy person | 1.65 (0.05) | -2.95 (0.09) | -1.72 (0.08) | 2.08 (0.09) |
|  | | | | |

^a^*λ* = factor loading estimate.

^b^*τ =* threshold estimate.

^c^Response categories of “limited a lot” and “limited a little” were combined due to low frequencies for items PF9 and PF10.

^d^Response options for all mental health sub-scale items were collapsed to four categories due to low frequencies for some categories.

*denotes a value that is not statistically significant at *α =* 0.05/10 = 0.005 for the PH sub-scale items or at α = 0.05/5 = 0.01 for the MH sub-scale items.
